# At the Limit of Interfacial Sharpness in Nanowire Axial Heterostructures

**DOI:** 10.1021/acsnano.4c04172

**Published:** 2024-07-06

**Authors:** Donovan Hilliard, Tina Tauchnitz, René Hübner, Isaak Vasileiadis, Athanasios Gkotinakos, George Dimitrakopulos, Philomela Komninou, Xiaoxiao Sun, Stephan Winnerl, Harald Schneider, Manfred Helm, Emmanouil Dimakis

**Affiliations:** † Institute of Ion Beam Physics and Materials Research, Helmholtz-Zentrum Dresden-Rossendorf, Dresden 01328, Germany; ‡ TUD Dresden University of Technology, Dresden 01062, Germany; § Department of Physics, Aristotle University of Thessaloniki, Thessaloniki 54124, Greece

**Keywords:** semiconductors, nanowires, vapor−liquid−solid, heterostructures, interfaces, GaAs, AlGaAs

## Abstract

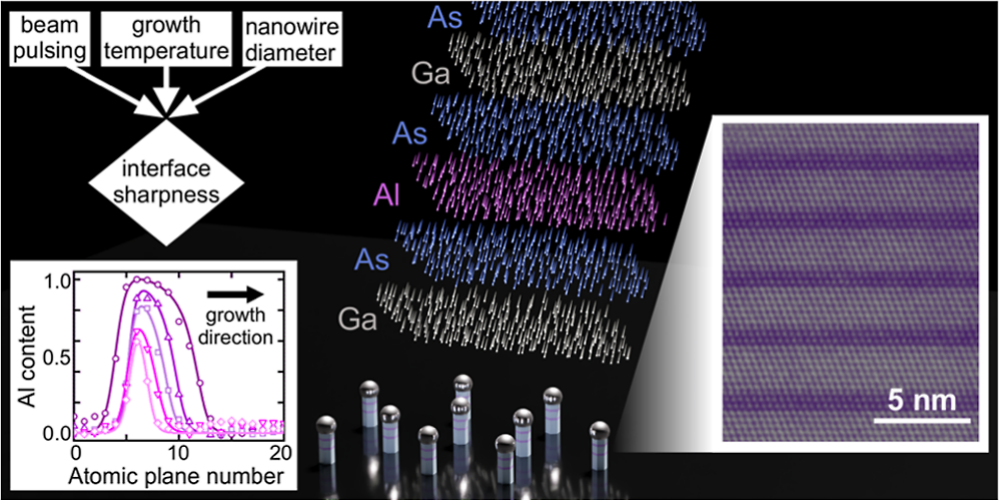

As semiconductor devices approach dimensions at the atomic scale, controlling the compositional grading across heterointerfaces becomes paramount. Particularly in nanowire axial heterostructures, which are promising for a broad spectrum of nanotechnology applications, the achievement of sharp heterointerfaces has been challenging owing to peculiarities of the commonly used vapor–liquid–solid growth mode. Here, the grading of Al across GaAs/Al_*x*_Ga_1–*x*_As/GaAs heterostructures in self-catalyzed nanowires is studied, aiming at finding the limits of the interfacial sharpness for this technologically versatile material system. A pulsed growth mode ensures precise control of the growth mechanisms even at low temperatures, while a semiempirical thermodynamic model is derived to fit the experimental Al-content profiles and quantitatively describe the dependences of the interfacial sharpness on the growth temperature, the nanowire radius, and the Al content. Finally, symmetrical Al profiles with interfacial widths of 2–3 atomic planes, at the limit of the measurement accuracy, are obtained, outperforming even equivalent thin-film heterostructures. The proposed method enables the development of advanced heterostructure schemes for a more effective utilization of the nanowire platform; moreover, it is considered expandable to other material systems and nanostructure types.

## Introduction

III–V semiconductor heterostructures have a central role with diverse functionality in electronic and optoelectronic devices. Implementing such systems in freestanding nanowires could further broaden the scope of potential applications such as the design of complex quantum heterostructures,^1−4^ the management of light in nanoscale,^5−7^ the engineering of strain^8,9^ or crystal structure,^10−13^ and the heterogeneous integration in technology-relevant platforms.^14−17^ It also raises expectations for on-demand photon sources (quantum dots^18−22^ and distributed Bragg reflectors^23−25^ hosted in the same nanowires) in quantum technology platforms. However, reducing the physical dimensions of heterostructures in nanotechnology devices toward atomic scales brings about a critical need for precise modulation of the chemical composition and effective management of the unintentional compositional grading across heterointerfaces. Any deviations from the intended compositional profile can in principle influence the heterostructure’s electronic properties, e.g. the emission photon energy^26−28^ or the photon entanglement^22^ in quantum dots, the transport and optical properties in quantum cascade lasers,^29^ or the electron and hole transport in transistors.^30^

Preferentially, III–V nanowires are grown in vapor–liquid–solid (VLS) mode,^31^ a bottom-up process where liquid droplets drive the epitaxial growth of monocrystalline nanowires on a substrate. VLS mode offers high degree of control over the nanowire crystal structure^10,12,13^ and dimensions,^32,33^ and affords the possibility to create axial, as well as radial, heterostructures using combinations of alloys with different chemical compositions.^8,9,34−40^ However, it is generally accepted that axial heterostructures cannot be very sharp when grown in VLS mode. Although compositional changes in the parent-vapor phase can be quite fast, they do not necessarily result in equally fast compositional changes in the middle-liquid and, eventually, the end-solid phases. This means that unintentional compositional gradients typically form across axial heterostructures, limiting the interfacial width to several atomic planes (hereafter referred to as monolayers, MLs) at best. Successful attempts have been made at improving the interfacial sharpness by employing thinner nanowires,^36,41,42^ lower growth temperatures,^36^ a higher Au concentration in Au-catalyzed nanowires,^43^ solid catalysts^44,45^ or flux pulsing^35,36^ (i.e., deposition of an element while the growth is temporarily interrupted). Among various material systems, GaAs/Al_*x*_Ga_1–*x*_As heterostructures are a distinctively attractive choice for device applications due to their tunable electronic properties in combination with a negligible lattice mismatch between the two materials. The challenge here lies in controlling the compositional grading of Al at the heterointerface. Using GaAs/Al_*x*_Ga_1–*x*_As/GaAs double heterostructures grown by conventional molecular beam epitaxy (MBE) methods (i.e., with a continuous supply of As) in self-catalyzed VLS mode (i.e., with Ga droplets driving the growth), Priante et al.^35^ demonstrated that the sharpness of the leading interface can be improved by lowering the As flux (i.e., lowering the growth rate) during the deposition of Al, achieving essentially a faster filling of the Ga droplet with Al. Their best result was a-few-MLs-thick interfaces obtained when the As flux was completely interrupted (along with the growth) during the supply of Al, a growth scheme that was termed droplet prefilling. In contrast, the second interface was always significantly thicker owing to the so-called reservoir effect,^35,36,41,42^ which in this case prevents the sharp transfer of Al atoms from the liquid into the solid phase. That is, Al atoms remain in the Ga droplet even though the supply of Al has been interrupted, and keep incorporating into the next several MLs of the growing solid creating a compositionally graded interface. This compositional grading has been described fairly well in a few cases using models based either on thermodynamic equilibrium at the interface between the liquid droplet and the solid nanowire^35^ or on nucleation theory,^46,47^ pointing toward a potential improvement only in thinner nanowires.

This work aims at exploring the limits of the interfacial sharpness in thin GaAs/Al_*x*_Ga_1–*x*_As/GaAs double axial heterostructures (Al_*x*_Ga_1–*x*_As insertions) in self-catalyzed VLS-grown GaAs nanowires. To this end, we present an extensive quantitative study of the Al grading as a function of the main experimental parameters, i.e., the nanowire radius (*R*_NW_), the growth temperature (*T*_G_), and the Al content (*x*). A key element of this work is the use of our previously developed growth technique called droplet-confined alternate pulsed epitaxy (DCAPE),^48^ an adaptation over conventional MBE. It employs only alternate pulses of Ga, Al, and As, which appears to be ideal for the droplet prefilling with Al. Furthermore, DCAPE provides precise control over the axial growth rate, the droplet composition, and the droplet contact angle, along with the opportunity to grow nanowire heterostructures at low-enough, Si-CMOS-compatible temperatures. Quantitative *x* profiles across the Al_*x*_Ga_1–*x*_As insertions are extracted from high-angle annular dark-field scanning transmission electron microscopy (HAADF–STEM) images and simulations following the methodological approach presented in ref (49). Building on an existing thermodynamic equilibrium growth model,^35^ we present a semiempirical function that describes well the *x* profile as a whole and allows for a rigorous analysis of both the profile’s leading and trailing interfaces. We show that the leading interfacial sharpness can be maximized in thin nanowires, largely due to the droplet prefilling possibilities provided by DCAPE. Most important, we demonstrate increases in trailing interfacial sharpness owing to modulation of the reservoir effect by decreasing the nanowire radius and/or the growth temperature. In the best case, practically symmetrical insertions with interface widths of only 2–3 ML, i.e., at the accuracy limit of our method, are achieved for the full range of *x*, reaching practically the absolute limit of atomically sharp interfaces and allowing for the realization of more complex heterostructures.

## Results and Discussion

### Growth and Compositional Analysis of Nanowire Heterostructures

**Figure 1:**
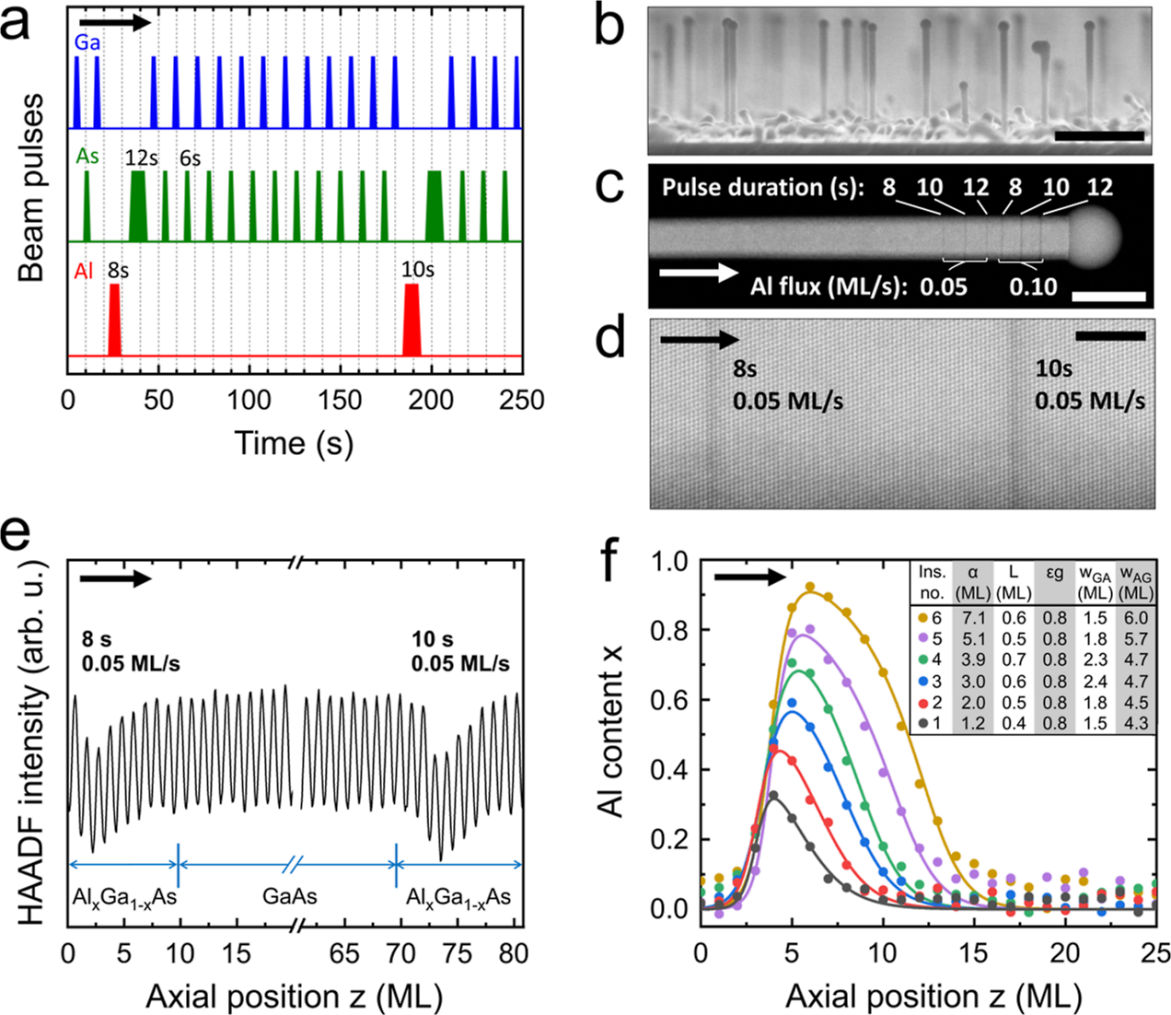
Overview of the Al_*x*_Ga_1–*x*_As insertion growth in GaAs nanowires and the compositional analysis. (a) Schematic depicting the sequence of alternating Ga (blue), As (green), and Al (red) pulses for GaAs/Al_*x*_Ga_1–*x*_As heterostructure growth. (b) Side-view SEM image of as-grown nanowires. (c) Low-magnification HAADF-STEM image of a nanowire containing 6 Al_*x*_Ga_1–*x*_As insertions of different Al contents visible by dark contrast. Labels show the corresponding Al pulse durations and fluxes. (d) Atomically resolved HAADF–STEM image of the bottom insertions: 8 and 10 s with 0.05 ML/s of Al. The scale bars in (b), (c), and (d) are 500, 100, and 5 nm, respectively. (e) Integrated HAADF intensity profile across the two insertions in (d), plotted as a function of position along the growth direction *z*. Oscillations are the intensity variations across individual ML. Larger dips in intensity indicate the presence of Al. (f) A series of Al-content profiles of insertions grown at 550 °C, calculated from the HAADF intensity and plotted as a function of the axial position *z*. The profiles are shifted along the position axis to overlap for a better comparison. The nanowire radius was 29 nm. Symbols represent experimental data, while the curves correspond to model fits. In this sample, the Al flux is 0.1 ML/s for insertions 1 to 6 with pulse durations of 2, 4, 6, 10, 16, and 24 s, respectively. The tabulated legend contains for each profile the insertion number in the nanowire (Ins. no.), the total amount of Al α, the interfacial sharpness quantifiers *L* and ε*g* for the leading and trailing interfaces, respectively, and the respective interface widths *w*_GA_ and *w*_AG_. Block arrows in (c–f) indicate the growth direction.

Self-catalyzed GaAs nanowires of zincblende (ZB) crystal structure containing thin Al_*x*_Ga_1–*x*_As insertions were grown in VLS mode on native-SiO_*x*_/Si(111) substrates by MBE, using the DCAPE method^48^ (see Methods–Growth). A typical sequence of beam pulses for heterostructure growth is demonstrated schematically in Figure 1a. First, atomic Ga and molecular As_4_ beams are delivered in alternating pulses with interruptions in-between to grow GaAs nanowire stems on the substrate, which is heated at 550 °C. Nanowire growth proceeds along the [111®] crystallographic direction (nanowire axis) and only during the As_4_ supply time. An example of the resultant nanowires can be seen in the SEM image of Figure 1b. The radius was deliberately controlled by varying the flux and/or the duration of the Ga pulses (see details in Methods–Growth). Second, by introducing a number of distant Al pulses to the sequence, an equal number of Al_*x*_Ga_1–*x*_As insertions were formed close to the top end of the nanowires. The heterostructure growth can be performed at either the same (550 °C) or a different temperature. The amount of Ga, Al, and As_4_ offered in each pulse is defined by both the flux and the duration of the pulse. The first As_4_ pulse after an Al pulse has a longer duration to ensure complete consumption of Al from the droplet. The axial growth rate is limited by the A_s4_ flux. A critical parameter of the growth is the droplet contact angle, which needs to remain in the range of 130–140° throughout the growth to ensure the formation of defect-free ZB nanowires.^12^ To this end, a balanced supply of group-III atoms and As_4_ to the droplet was achieved after a careful tuning of the respective pulses in separate test experiments. Additionally, as a precaution, a Ga pulse was offered before every insertion.

An example of a heterostructure is given in the low-magnification HAADF–STEM image of Figure 1c. Dark contrast lines denote 6 Al_*x*_Ga_1–*x*_As insertions. In this specific example, both the duration and the flux of the Al pulses were varied (the duration is given in seconds above and the flux in ML/s below). An atomically resolved portion of the image showing the bottom two insertions, i.e. 8 and 10 s pulses with a 0.05 ML/s Al flux, is shown in Figure 1d, demonstrating the capability of DCAPE in growing defect-free heterostructures (a few stacking faults and rotational twin planes were observed in some cases and are attributed to random growth fluctuations). The corresponding intensity profile along the growth direction (see Methods–HAADF–STEM: Experimental Measurement of Al-Content Profiles) is plotted in Figure 1e. Each local intensity peak corresponds to one (111) atomic plane of group-III atoms normal to the growth direction, whereas the several-ML-thick intensity dips (two of them are shown in Figure 1e) indicate the incorporation of Al. Such intensity profiles are converted to Al-content profiles using HAADF image simulations assuming random Al_*x*_Ga_1–*x*_As alloys and taking into account the nanowire thickness and electron-channeling and beam-broadening effects (see Methods–HAADF–STEM: Experimental Measurement of Al-Content Profiles). A representative example is shown in Figure 1f, where the ML-resolved Al profiles of all 6 insertions grown at 550 °C inside a nanowire with a radius of 29 nm are plotted (shifted along the position axis to overlap for a better comparison). We note that the nanowire radius in all studied samples remains constant along the segment with the 6 insertions, and typically it is measured at the end of that segment, just below the Ga droplet (see Figure S1 in Supporting Information). As seen, a wide range of peak Al contents *x*_0_ from 0.3 to almost 1 was obtained, demonstrating the ability to deliberately change the insertion composition. More details about growth control and reproducibility can be found in the Supporting Information (Figure S2). Interfaces going in the GaAs-to-Al_*x*_Ga_1–*x*_As and Al_*x*_Ga_1–*x*_As-to-GaAs directions along the growth direction are hereafter referred to as the GA and AG interfaces, respectively. It is evident that *x* rises fast (GA interface) in all insertions, reaching its peak value after 3–4 ML, and then drops with a slower rate (AG interface), which is indicative of the reservoir effect. The error in *x* measurements is in the range of ±0.05 and originates from the image quality (scanning and random shot noise) and the chemical inhomogeneity along a ML (see Figure S3 in Supporting Information). Due to the unavoidable electron cross-scattering from adjacent MLs in HAADF–STEM measurements, image simulations show that it is challenging to reliably determine interfacial compositional profiles with resolution better than 2 ML (see Methods–HAADF–STEM: Experimental Measurement of Al-Content Profiles and Figure S4 in Supporting Information).

For a systematic study of the compositional grading across Al_*x*_Ga_1–*x*_As insertions, two sample series were grown (see Methods–Growth). The first series consists of three samples with the same heterostructures (6 Al_*x*_Ga_1–*x*_As insertions with different *x*) grown at different *T*_G_ (350, 450, and 550 °C) on GaAs stems with *R*_NW_ = 20–25 nm. The second series consists of four samples with the same heterostructures, all grown at *T*_G_ = 550 °C, on GaAs stems with different *R*_NW_ from 9 to 65 nm. With the exception of one sample in which Al was delivered according to Figure 1a (varying both pulse duration and flux), for all other samples, the Al pulse durations for the insertions 1 to 6 were 2, 4, 6, 10, 16, and 24 s, respectively, delivered with a flux of 0.1 ML/s. Fluctuations from the nominal Al content may be attributed to unforeseen changes in the droplet contact angle during growth, but are more likely the result of random growth fluctuations along the substrate surface.

### Fitting the Al Profile with a Semiempirical Function

For a quantitative description of Al grading across the Al_*x*_Ga_1–*x*_As insertions in self-catalyzed GaAs nanowires, we developed a semiempirical model based on a preexisting model proposed by Priante et al.^35^ According to the latter, the liquid–solid Ga–Al–As system is considered to be at thermodynamic equilibrium. Although, in reality, growth takes place out of equilibrium, the model was able to successfully reproduce the experimental data of the Al grading, being also in good agreement with alternative nucleation models.^46,47^ Given that the VLS growth proceeds in a periodic ML-by-ML fashion (as a result of the low solubility of As in liquid Ga),^12^ it is presumed that the solid Al content *x* in each ML is exclusively defined by the liquid Al concentration *y* in the Ga droplet just before the formation of that ML. The relation between *x* and *y* at a given temperature, i.e. the ternary solidus isotherm,^50^ can be approximated by the simple expression^35,51^
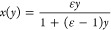
1

**Figure 2:**
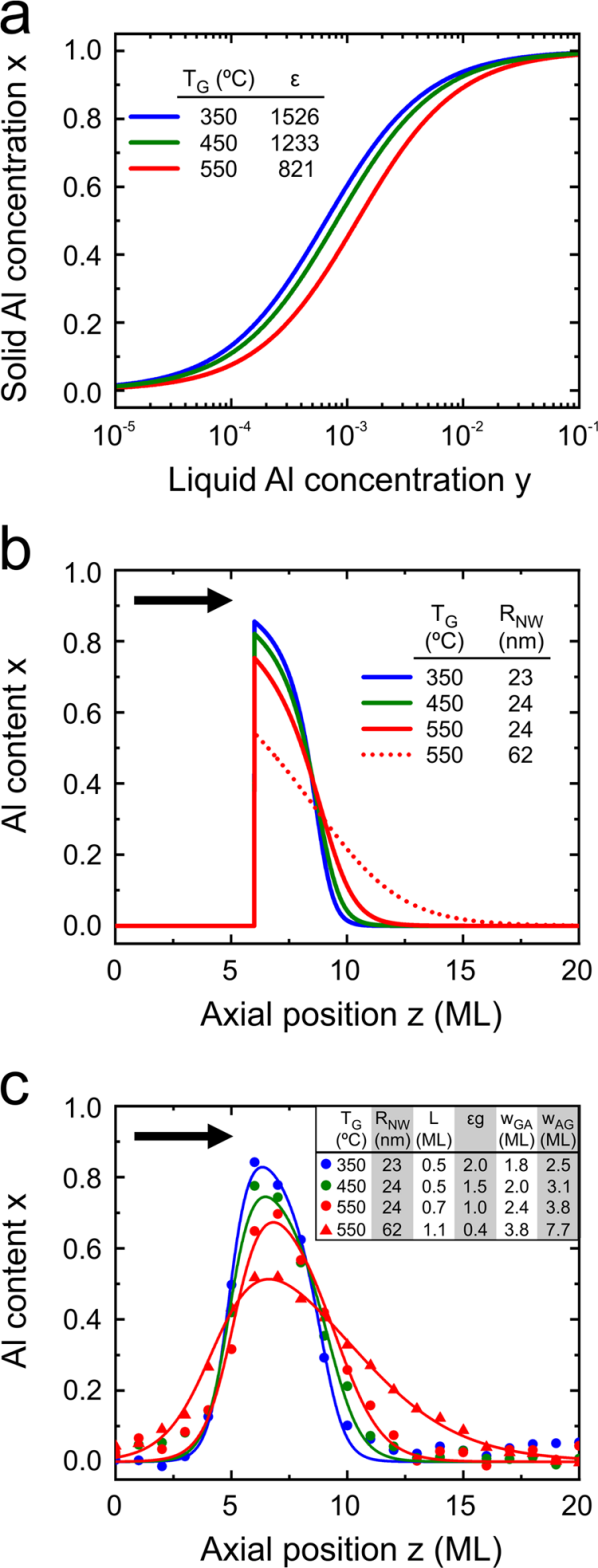
Al-content profile shape across Al_*x*_Ga_1–*x*_As insertions grown at different temperatures (*T*_G_) or in nanowires with different radii (*R*_NW_). (a) Simulated ternary solidus isotherms for the Ga–Al–As system at *T*_G_ = 350 (blue), 450 (green), and 550 °C (red), using eq 1 with ε = 1526, 1233, and 821, respectively. (b) Al-content profiles simulated with the thermodynamic model outlined in ref (35) for the corresponding growth temperatures given in (a). For all profiles shown, the same total Al content of α = 2 ML was used, along with the same droplet contact angle β = 140°. Solid and dotted lines represent profiles with *R*_NW_ = 23–24 and 62 nm, respectively. (c) Experimental Al-content profiles for insertions containing α ≈ 3.5 ML of Al, grown at the corresponding growth temperatures given in (a) and (b). Symbols and curves represent experimental measurements and model fits with eq 4, respectively. The basic profile and fit parameters are listed in the table inset. Block arrows in (b) and (c) indicate the growth direction.

The quantity ε (≫ 1) depends only on temperature and denotes the ratio of the crystallization rates of the Al^l^ + As^l^ → AlAs^s^ and Ga^l^ + As^l^ → GaAs^s^ reactions (l and s superscripts stand for liquid and solid phase, respectively). We infer from ref (47) that ε ≈ 821, 1233, and 1526 for our insertions grown at 550, 450, and 350 °C, respectively. The corresponding solidus isotherms are plotted in Figure 2a. Generally, any solid Al content *x* requires only a low liquid Al concentration *y* (i.e., *y* ≪ *x*). The decrease in temperature (increase of ε) shifts the isotherm curve in a way that the same *x* values can be obtained with a lower *y*. It is shown in the following that this is beneficial for the AG interfacial sharpness.

The growth of a nanowire ML with solid Al content *x* is accompanied by a decrease in liquid Al concentration *y*, which is given by the following mass balance equation^35^
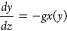
2where *z* is the axial coordinate in ML along the growth direction, and *g* is a geometrical factor that is equal to the number of group-III atoms in a ML of a nanowire with radius *R*_NW_, divided by the number of all atoms in the liquid droplet (practically only Ga) with contact angle β (see the analytical expression of *g* in Methods–Model equations). Equation 2 has an analytical solution for *y*(*z*), which can be plugged into eq 1 to get the Al-content profile of the Al_*x*_Ga_1–*x*_As insertion^35^
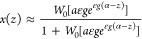
3where *W*_0_ is the principal branch of the Lambert function,^52^ and α is the total amount of Al (given in numbers of equivalent AlAs atomic planes, i.e. in numbers of ML, in the nanowire) supplied by one Al pulse to the Ga droplet and eventually incorporated in the insertion (*y*_0_ = α*g* gives the initial value of *y*, i.e. after the Al pulse and before the following As_4_ pulse).

According to the thermodynamic equilibrium model alone, the Al profile from eq 3 should have the form shown in Figure 2b for three different growth temperatures *T*_G_ (continuous curves with different colors) and two different nanowire radii *R*_NW_ (continuous and dotted red curves), for the same total Al content α = 2 ML. The abrupt GA interface is a consequence of the droplet prefilling with Al (i.e., Al is supplied while the growth is temporarily interrupted), so that each insertion begins with the highest value of *y* and, thus, the highest value of *x*. However, the experimental data in Figure 1f clearly show that this is not the case. Instead, the GA interface always has a finite thickness that exceeds the experimental accuracy of 2 ML, with the rise of *x* exhibiting a sigmoidal behavior. Such sigmoidal compositional grading is very common in vapor–solid (VS)-grown GaAs/Al_*x*_Ga_1–*x*_As thin-film heterostructures (as well as in other binary/ternary alloy heterostructures), denotes that the incorporation of the inserted element (Al in this case) depends on its content in the underlying ML, and has been correlated with a 2D-island-mediated growth mode.^53^ We could thus presume that the Al incorporation also in VLS-grown nanowire heterostructures depends on the Al content of the underlying ML. However, the exact accountable mechanism is unknown, and we cannot rule out at this point that the sigmoidal profile has different origins. In our analysis, we consider only flat interfaces as a consequence of the ML-by-ML growth mode [see Methods–HAADF–STEM: Experimental Measurement of Al-Content Profiles].

In any case, irrespective of the actual underlying mechanisms, the sigmoidal shape of the GA interface can be reproduced by multiplying the right-hand side of eq 1 by a *z*-dependent generalized logistic function (*C* + *e*^(*z*_0_–*z*)/*L*^)^−1^.^54,55^*C* defines the upper-right asymptote (the lower-left asymptote is zero) and *L* represents the width of the sigmoidal function, which is centered at *z*_0_. Solving eq 2 with the new *x*(*y*) relation, the Al-content profile of an Al_*x*_Ga_1–*x*_As insertion is now approximated (see full function in Methods–Model equations) as
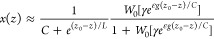
4where γ = αε*ge*^αε*g*^(*e*^–*z*_0_/*L*^ + 1/*C*)^ε*gL*/*C*^. The sharpness of the GA interface is simply quantified by the interface parameter *L* (the lower its value, the sharper the interface), which is independent of the Al composition, whereas the AG interfacial sharpness is quantified by the factor ε*g*/*C* (the higher its value, the sharper the interface). Here, εg represents the contribution of the reservoir effect alone, and *C* any deviation from it (in a simplest approximation). This means that the AG interfacial sharpness is limited by the reservoir effect if *C* = 1, whereas the interface is broader or sharper if *C* > 1 or <1, respectively.

Figure 1f shows with continuous curves that our semiempirical function successfully fitted the experimental data sets of all insertions, describing well both the GA and the AG interfaces. The parameter values of the fits are listed in the inset. For every fit, the values of ε and *g* are set according to the given *T*_G_ and *R*_NW_, respectively, whereas the values of α, *L*, and *C* are deduced as fit parameters (β = 140° was assumed in all cases). Within the statistical error, the value of *C* was always approximately equal to 1, manifesting that the sharpness of the AG interface is indeed limited by the reservoir effect as described by the thermodynamic equilibrium model (see Supporting Information for all measured insertions in Figure S5: *C* vs *R*_NW_ with α gradient). Deviations with *C* > 1 are consistently observed only in insertions that have both low α and *R*_NW_ values. However, we interpret this as an artifact that stems from the low signal-to-noise ratio in HAADF–STEM measurements (these insertions are excluded from the following analyses).

In addition to *L* and ε*g*/*C*, for a common and more perceptible quantifier of both interfaces, we use the commonly reported interfacial width *w* (in ML), over which the insertion’s Al composition changes from 10 to 90% or from 90 to 10% of its peak value *x*_0_. The widths of the Al_*x*_Ga_1–*x*_As insertions in Figure 1f are listed therein: *w*_GA_ and *w*_GA_ denote the widths of the GA and AG interfaces, respectively.

### Dependence of Interfacial Sharpness on Growth Parameters

To study the effect of the main experimental parameters on interfacial sharpness, we used the semiempirical function of eq 4 to fit the Al profiles of insertions with different total supplied Al contents α, grown at different temperatures *T*_G_, and in nanowires with different radii *R*_NW_. A vast number of insertions, 145 in total (typically extracted over 5 nanowires per sample), were measured and analyzed. To visually demonstrate the fit quality and effect of varying *T*_G_ and *R*_NW_, a representative subset of Al_*x*_Ga_1–*x*_As profiles, each comprising a similar total supplied Al content (α ≈ 3.5 ML), is presented in Figure 2c. Different colors and symbol shapes represent different *T*_G_ and *R*_NW_, respectively. In accordance with the calculations in Figure 2b, we observe a clear sharpening of the AG interface with decreasing either *R*_NW_ or *T*_G_ for a constant α. This is expressed by an increase of ε*g*/*C* (*C* ≈ 1) and a decrease of *w*_AG_ (all values as deduced from each fit are listed in the inset), as well as by an increase of *x*_0_. Concurrently, we also observe a sharpening of the GA interface, which is expressed by a decrease of both *L* and *w*_GA_. We note that the value of α in the calculations in Figure 2b was set approximately equal to the amount of Al within the AG interface in Figure 2c (≈2 ML), so that a straight comparison between calculated and experimental profiles of the AG interface is possible.

**Figure 3:**
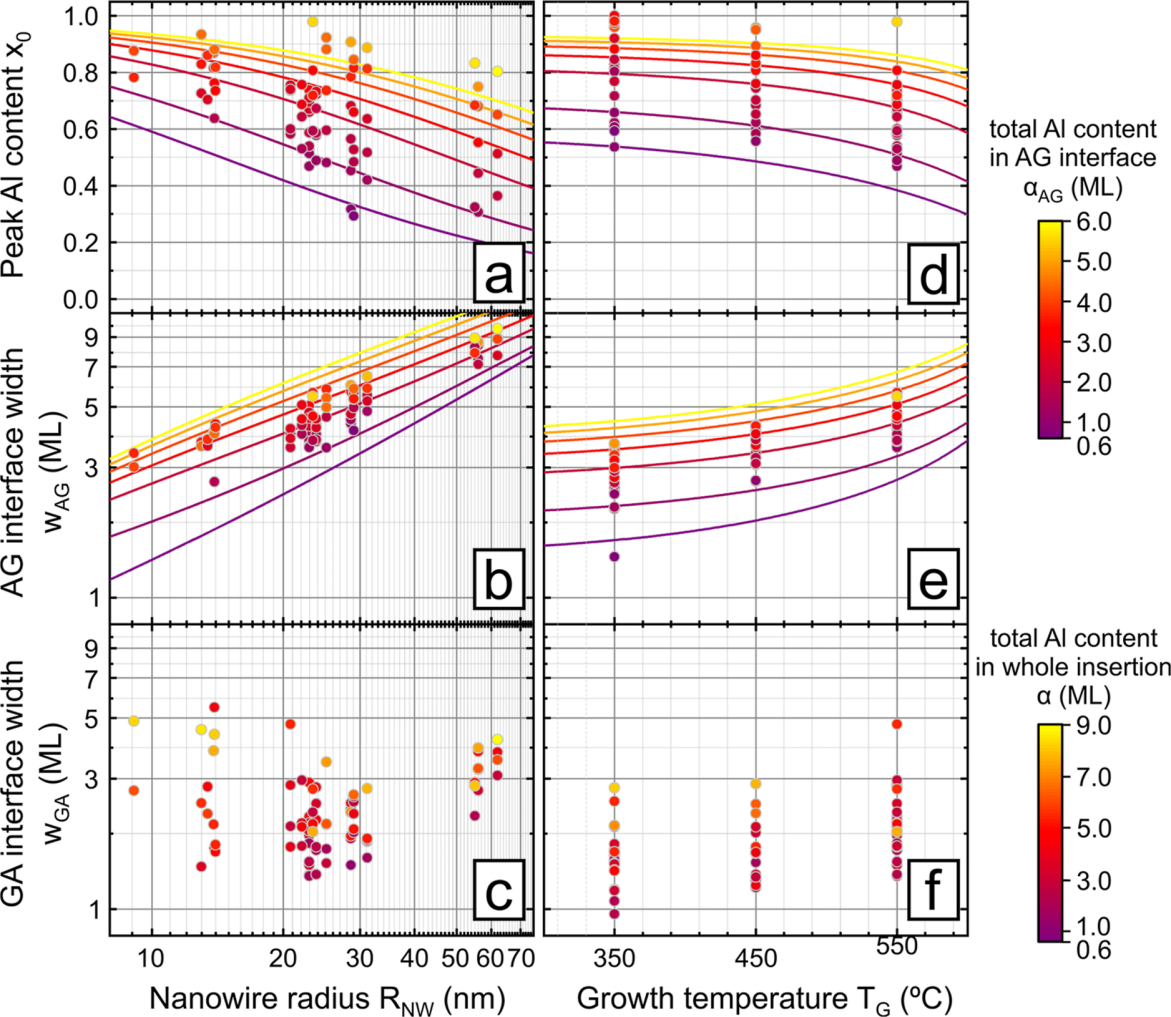
Results summary for all measured Al_*x*_Ga_1–*x*_As insertions showing a consistent improvement of the Al-content profile sharpness at smaller nanowire radii (*R*_NW_) and lower growth temperatures (*T*_G_). The GaAs-to-Al_*x*_Ga_1–*x*_As and Al_*x*_Ga_1–*x*_As-to-GaAs interfaces are denoted as GA and AG, respectively. Plots of the Al-profile peak (*x*_0_) and interface widths (*w*_AG_ and *w*_GA_) as a function of *R*_NW_ for *T*_G_ = 550 °C (panels a–c), or as a function of *T*_G_ for samples with *R*_NW_ = 20–25 nm (panels d–f). Symbols and solid lines represent experiment and theory with eq 3, respectively. The solid lines in (d, e) were calculated for an average *R*_NW_ = 23 nm. The color gradient represents the total Al contained either in the AG interface (α_AG_) in (a,b,d,e) or in the whole Al_*x*_Ga_1–*x*_As insertion (α) in (c, f).

The complete set of measured peak values *x*_0_ and widths *w*_GA_ and *w*_AG_ is plotted as a function of the nanowire radius *R*_NW_ in Figure 3a–c (for all insertions grown at *T*_G_ = 550 °C) and the insertion growth temperature *T*_G_ in Figure 3d–f, (for all insertions with similar radii *R*_NW_ = 20–25 nm). The symbols depict the experimental data, whereas the solid lines are the theoretical values as described by the reservoir effect for the AG interface alone. Each line was calculated with eq 3 for a different total Al content within the AG interface (as deduced from the fit of the experimental data with eq 4), whereas an average *R*_NW_ = 23 nm was used for Figure 3d,e. All data in the plots use a color gradient that represents the total Al (in ML) contained either in the AG interface (termed as α_AG_ in Figure 3a,b,d,e) or in the whole Al_*x*_Ga_1–*x*_As insertion (termed as α in Figure 3c,f).

In good agreement with theory, the experimental data clearly show a consistent sharpening of the AG interface of all insertions (i.e., an increase of *x*_0_ and a decrease of *w*_AG_) with decreasing either *R*_NW_ or *T*_G_ for a fixed total Al content. Also the GA interface, which is anyway quite sharp (*w*_GA_ < 5 ML), shows a slight improvement with decreasing either *R*_NW_ or *T*_G_ for insertions with α < 7 ML. Here, we need to point out that *w*_GA_ values lie at, or below, the resolution limit of our STEM methodology and should be seen as indicative only. Concerning *x*_0_, this increases in accordance with theory by increasing the total Al content at fixed *R*_NW_ and *T*_G_, at the expense though of both interfacial widths. However, the profile slopes remain the same for a fixed *R*_NW_ (see *L* and εg in Supporting Information Figure S6), which means that the increase of both *w*_GA_ and *w*_AG_ with increasing the total Al content stems naturally from the fact that it takes more MLs to change *x* both from 0 to *x*_0_ and from *x*_0_ back to 0 when *x*_0_ is larger. This explains why the effect of *R*_NW_ and *T*_G_ on *w*_GA_, which is weak regardless, is obscured for insertions with α > 7 ML. After all, the sharpest insertions are obtained for a combination of *R*_NW_ = 20–25 nm and *T*_G_ = 350 °C, reaching *w*_GA_ and *w*_AG_ values as low as approximately 1–4 ML for *x*_0_ up to 1.

For the AG interfaces, the good agreement between experiment and theory means that the thermodynamic equilibrium model can sufficiently describe the incorporation of Al from the liquid into the solid phase (on the basis of the reservoir effect), without any obvious influence by other reported growth mechanisms, such as the limited diffusivity of Al and As in the liquid,^56^ the periodic mass transfer (including Al) from the fluctuating edges of the nanowire tip to the liquid,^12^ or the atomic ordering on the liquid side of the liquid–solid interface.^57^ In contrast, the thermodynamic equilibrium model alone (without the addition of the empirical sigmoidal function) failed to describe the sigmoidal shape of the GA interfaces. The actual GA profile is smeared out by the electron cross-scattering effect, but the observed dependence on *R*_NW_ and *T*_G_, although weak, probably implies the existence of an underlying compositional grading mechanism. At this stage, we can only speculate about possible grading mechanisms, which could involve, for example, the unintentional growth during the droplet prefilling with Al (owing to the presence of As_4_ in the background), the substitutional synthesis as it has been reported for metal-rich growth of In_*x*_Ga_1–*x*_N,^49^ the interdiffusion of Ga and Al atoms (although it seems unlikely at this range of low temperatures), or any combination of the above.

**Figure 4:**
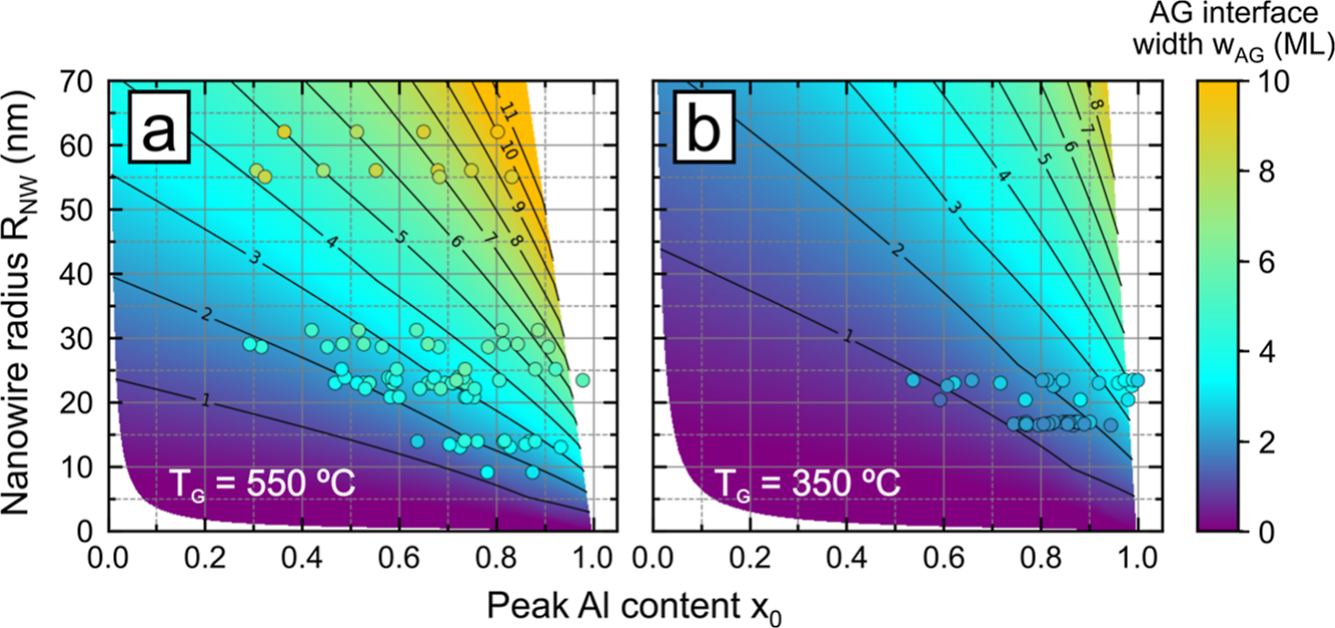
Summary maps showing the increased feasibility of sharp Al_*x*_Ga_1–*x*_As-to-GaAs (AG) interfaces at lower growth temperatures (*T*_G_). The color maps show the AG interface width (*w*_AG_) as a function of the nanowire radius (*R*_NW_) and the peak Al content (*x*_0_) for our upper and lower *T*_G_ extremes of 550 °C (panel a) and 350 °C (panel b). Symbols and shaded areas respresent experiment and theory with eq 3, respectively. The black lines are *w*_AG_-isolines for the correspondingly labeled values in ML.

In designing heterostructures to tailor their (opto)electronic properties, what matters the most is having access to the whole range of compositions (for Al_*x*_Ga_1–*x*_As in this case) with a minimum interfacial width. As demonstrated here, the droplet prefilling with Al achieves exceptionally narrow GA widths for any composition *x*, but the AG width, due to the reservoir effect, depends strongly on the nanowire radius and the growth temperature. As summarized in Figure 4a, both theory (color map) and experiment (data points) show that the nanowire radius must be small enough to keep the AG width small, especially for higher values of *x*_0_. However, this condition is waived at lower growth temperatures (Figure 4b). For example, insertions in the whole compositional range (*x*_0_ up to 1) with *w*_AG_ < 3 ML need nanowires with *R*_NW_ < 20 nm at 350 °C, which is more feasible than *R*_NW_ < 10 nm at 550 °C. Thus, the low growth temperature obviously offers more freedom in the design of heterostructures.

Overall, our results demonstrate that axial heterostructures in VLS-grown nanowires can be sharp, in contrast to what has been commonly ascertained. A direct comparison with other growth methods reported in the literature is difficult because of differences in crystallographic orientation, interfacial roughness, measurement methodology, probed interfacial area size, and growth conditions. Nevertheless, it is instructive to mention that state-of-the-art VS-grown GaAs/Al_*x*_Ga_1–*x*_As(001) thin-film heterostructures (including quantum cascade laser structures) with *x* in the range of 0.3–0.4 exhibit interfacial widths of 3–4 ML,^58,59^ which is among the sharpest binary/ternary alloy interfaces quoted in the literature. Impressively, our pulsed VLS growth mode resulted in comparable or even sharper axial interfaces in nanowires, highlighting the importance and the prospects of our findings.

### Profile Symmetry

**Figure 5:**
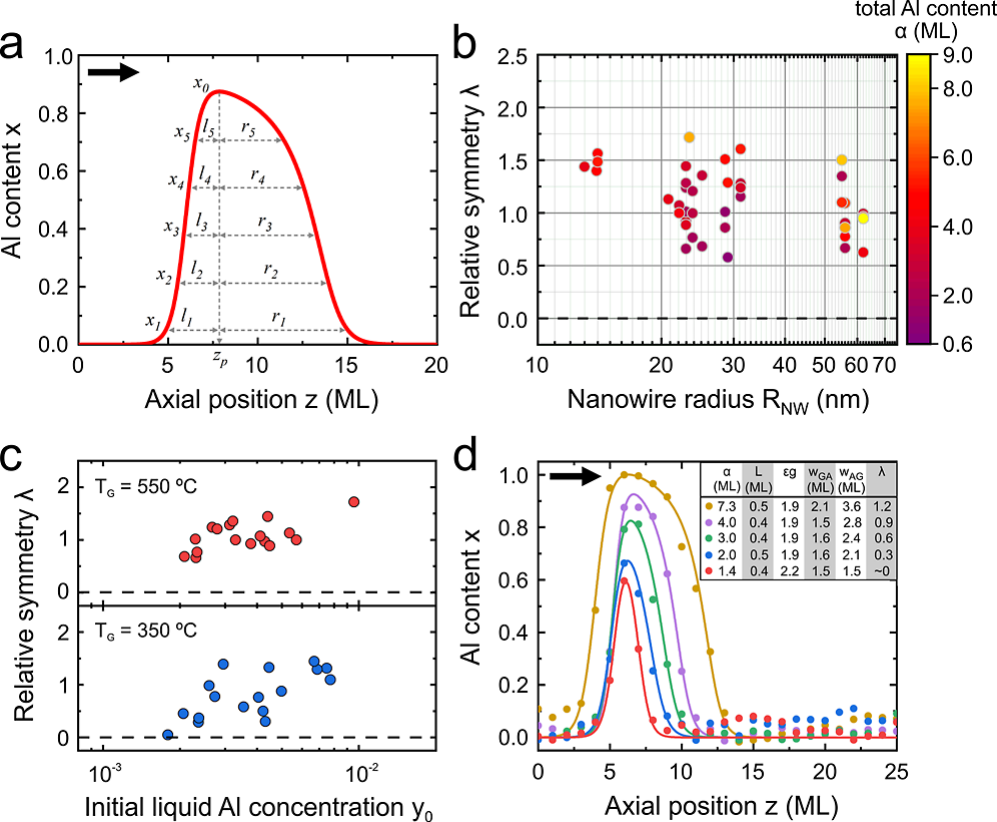
Analysis of the Al-content profile symmetry across Al_*x*_Ga_1–*x*_As insertions showing improvement at lower growth temperatures (*T*_G_) and lower Al contents (*a*). (a) Simulated Al-content profile that exemplifies the symmetry analysis methodolgy. The profile is analyzed at 5 equidistant Al-content levels *x*_*i*=1–5_ between *x*_1_ = 0.05 and the peak value *x*_0_. *l*_*i*=1–5_ and *r*_*i*=1–5_ are the corresponding widths on the left and right side, respectively, of the profile peak position *z*_P_. The relative symmetry (λ) is calculated with eq 5. (b) Plot of λ as a function of the nanowire radius (*R*_NW_) for insertions grown at *T*_G_ = 550 °C. The color gradient represents α. (c) Plots of λ as a function of the initial Al concentration (*y*_0_) in the droplets at *T*_G_ = 550 (upper panel) and 350 °C (lower panel) in nanowires with *R*_NW_ = 20–25 nm. The black dashed lines at λ = 0 in (b, c) denote perfect profile symmetry. (d) Experimental Al-content profiles for insertions grown at *T*_G_ = 350 °C with different total Al contents α. Symbols and curves represent experimental measurements and model fits with eq 4, respectively. The corresponding fit parameters and λ values are listed in the table inset. The block arrows in (a) and (d) indicate the growth direction.

In this section, we analyze the symmetry of the Al profiles around their peak values. The extent of symmetry is expressed by the parameter
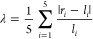
5where *l*_*i*_ and *r*_*i*_ are the widths of the Al-profile curve at selected equidistant values *x*_*i*_ (*i* = [1, 5]), left and right of the peak position, respectively. Figure 5a exemplifies the symmetry analysis methodolgy using a simulated Al profile with the parameters εg = 1.72, *L* = 0.4, and *a* = 6 ML. Profiles with such a combination of parameters tend to be skewed to the right and typically resemble those of insertions grown at 550 °C in our experiments. *x*_0_ denotes the profile’s peak Al value and *z*_p_ its position along the growth axis. *x*_*i*_ = *x*_1_ + (*i* – 1)(*x*_0_ – *x*_1_)/5, whereas *x*_1_ = 0.05 was chosen for all profiles based on the approximate upper noise threshold of our experimental profiles.

A plot of λ as a function of *R*_NW_ for *T*_G_ = 550 °C is shown in Figure 5b. The data point color gradient represents a scale for α, and λ = 0 (highlighted by the dashed line) corresponds to perfect profile symmetry, which is clearly not the case for these insertions. Interestingly, λ appears to remain almost constant throughout the entire *R*_NW_ range (for a fixed α), indicating that the improvement of both GA and AG interfaces with decreasing *R*_NW_ (see Figure 3) is such that the profile asymmetry does not noticeably change. Irrespective of *R*_NW_, though, profiles with low α tend to be more symmetrical. Such behavior is expected as a result of the formation of an asymmetrical plateau at higher total Al contents (see Figure 1f), which originates from the slow change of *x* at high *y* (see Figure 2a). On the contrary, profiles with low total Al contents tend to peak rather than plateau, exhibiting better symmetry.

The improved Al-profile symmetry at lower Al contents is also shown in Figure 5c for insertions with the same *R*_NW_ = 20–25 nm, grown either at 550 (red symbols) or at 350 °C (blue symbols). Here, λ is plotted as a function of the initial Al concentration in the liquid droplet *y*_0_ for a better comparison with the solidus isotherms in Figure 2a. Our results also show that a better profile symmetry is obtained at 350 °C, reaching λ values close to 0 when *y*_0_ < 4 × 10^–3^. In practice, very symmetrical (λ < 0.5) and sharp (*w*_GA_ and *w*_AG_ of around 2–3 ML) Al profiles with *x*_0_ up to 0.8 can be obtained at 350 °C. Figure 5d shows a set of five example profiles with *x*_0_ ranging from 0.6 up to 1. Here, it is demonstrated well that plateau effects become the dominant source of asymmetry in profiles where *x*_0_ > 0.8. Yet, the utility of insertions with *x*_0_ > 0.8 should not be dismissed. If we disregard the Al variation at the plateau (about 10–20%), both the GA and AG interfaces may be considered to be practically symmetrical with comparable interface widths of 2–3 ML. Of course, restrictions on profile symmetry may vary by application, but it stands to reason that all insertions with high Al contents grown at 350 °C are much more symmetrical when compared to their 550 °C-grown equivalents.

### Prospects

**Figure 6:**
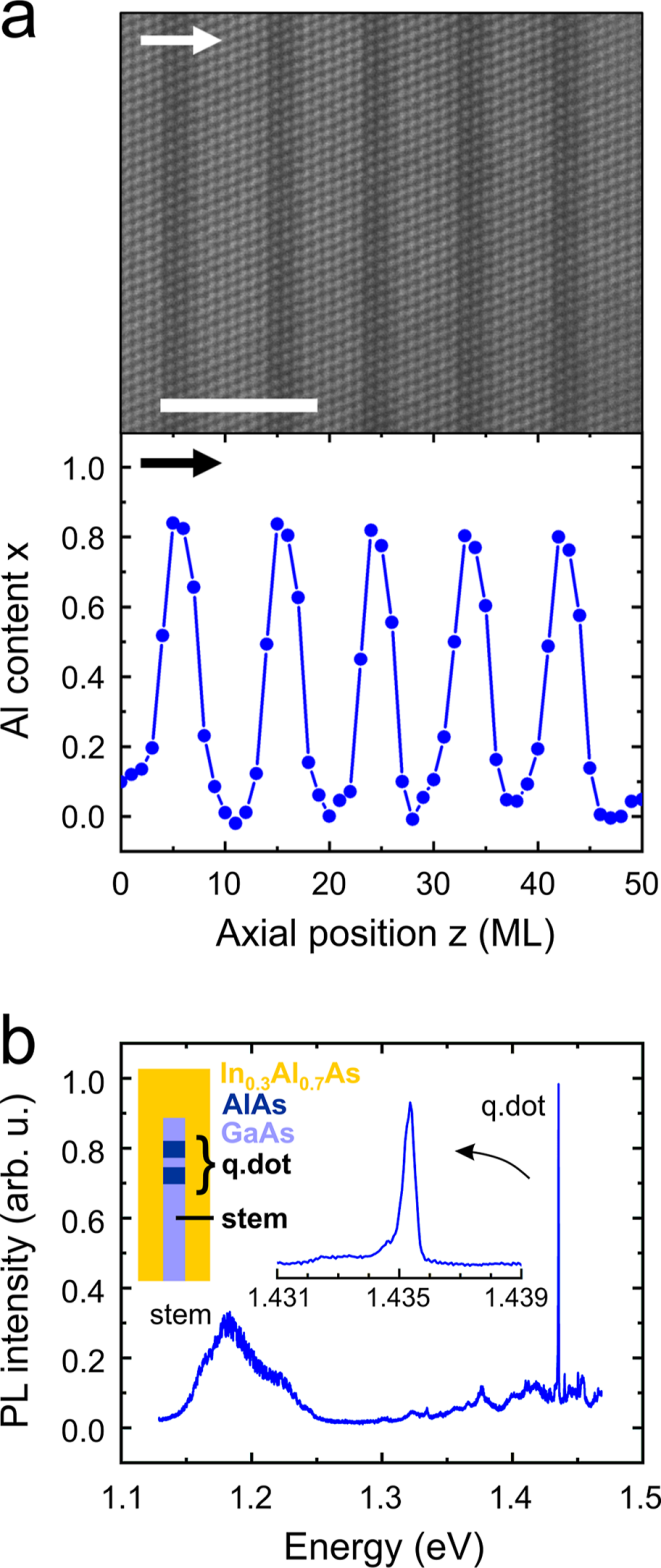
Complex heterostructures that exemplify the prospects of the proposed growth strategy. (a) A GaAs/Al_0.8_Ga_0.2_As short-period superlattice demonstrates the high degree of control over structural quality, thicknesses, and Al-content profiles that our pulsed growth method provides. Top panel: atomically resolved HAADF–STEM image of a short-period superlattice grown at a temperature of *T*_G_ = 350 °C in a nanowire with a radius of *R*_NW_ = 16 nm. The scale bar is 5 nm. Bottom panel: resultant Al-content profile. Top and bottom panels have the same horizontal scale. The block arrows indicate the growth direction. (b) μ-PL spectrum at 5 K from a single dot-in-wire. The inset shows a schematic description of the nanowire heterostructure. Emissions from the GaAs stem and the GaAs quantum dot (q.dot) are labeled accordingly. A close-up of the emission from the quantum dot is also shown.

The high structural quality and the accurately controlled thicknesses and composition profiles make our method suitable for the realization of complex heterostructure schemes, such as digital alloys for tuning the bandgap or forming quantum dots (single dots or dot chains), distributed Bragg reflectors, quantum cascade lasers, resonant tunneling diodes, etc. Here, we show an example of a short-period superlattice (i.e., an ensemble of closely spaced insertions) in nanowires with *R*_NW_ = 16 nm, grown at *T*_G_ = 350 °C. The high-resolution HAADF–STEM image and the corresponding Al profile in Figure 6a show that besides the absence of stacking faults and the apparent interfacial sharpness of only 2–3 ML, the heterostructure exhibits very good periodicity with ML-precision in thickness and identical Al profiles for all insertions.

Conveniently enough, the sharpest interfaces in our work were obtained at growth temperatures that fall within the thermal budget of fully processed Si-CMOS platforms, offering a realistic path for adaptation in semiconductor foundries, overcoming a serious compatibility obstacle in the realization of hybrid III–V/Si more-than-Moore systems. On the other hand, though, low growth temperatures typically account for the formation of point defects (possibly As vacancies and Ga antisites owing to the excess of Ga in the liquid phase^60^) and the incorporation of impurities (e.g., carbon or oxygen; although the excess of Ga at the growth interface might promote the formation of volatile GaO_*x*_, prohibiting the incorporation of oxygen). This is something that could negatively affect the sought-after semiconductor properties, such as the optical properties, and should certainly be contrasted with the benefits of growth at low temperatures (i.e., sharper interfaces and Si-CMOS compatibility). This subject needs to be addressed in detail in a separate study. Here, we only exemplify the high potential of our pulsed growth mode at low temperatures with the demonstration of light emission from a dot-in-wire heterostructure grown at 350 °C. The nanowire consists of a 30 nm-thick GaAs core with two 5 nm-thick AlAs axial insertions (axial quantum barriers) separated by 3 nm, and a 40 nm-thick In_0.3_Al_0.7_As shell for radial quantum confinement. A schematic of the structure is given as an inset in Figure 6b, whereas the growth details are given in Methods–Growth. A microphotoluminescence (μ-PL) spectrum at 5 K from a single nanowire is shown in Figure 6b (see Methods–μ-PL Measurements), where one can distinguish a broad emission at around 1.18 eV from the GaAs stem (i.e., the core without the quantum dot) and a sharp emission at 1.435 eV from the GaAs quantum dot. We note that the lattice-mismatched shell (In_0.3_Al_0.7_As has a 2.1% larger lattice constant than GaAs) induces tensile strain in the core, which narrows the bandgap of the core and, thus, red-shifts the light emission from both the stem and the quantum dot.^8^ Although it is not a decisive study, the quantum dot emission exhibits promising characteristics despite the low growth temperature: the line shape is relatively sharp (full width at half-maximum of 387 μeV) and the intensity is relatively high, even though part of the light is absorbed inside the stem.

It is evident that every material system possesses distinctive growth properties in terms of thermodynamics and kinetics, and thus, growth strategies are typically applicable (with modifications) to a select group of systems. Our strategy is likely to be applicable to other self-catalyzed material systems that have similar solubility relations to the Al–Ga–As system, without excluding, though, systems with foreign catalysts (e.g., Au). Self-catalyzed systems are of course technologically significant, as they avoid the use of foreign elements that could potentially act as impurities.^61^ What limits the reservoir effect and leads to sharper interfaces at lower growth temperatures is the enhanced propensity of the added element to leave the liquid for the solid phase, i.e. the decrease of the *y*/*x* ratio. This is also true for other self-catalyzed ternary systems of the broad (In, Al)-Ga-(As, P, Sb) semiconductor family,^47,62,63^ offering a wide range of band-structure engineering possibilities. In the case of lattice-mismatched heterostructures, the effect of elastic strain on the interface sharpness is an additional important topic that has not been studied systematically in nanowires and a future study would certainly be relevant.

## Conclusions

Using a pulsed VLS growth mode, we have obtained defect-free Al_*x*_Ga_1–*x*_As insertions in self-catalyzed GaAs nanowires on Si substrates, in the whole range of Al contents. The use of alternate pulses of Al, Ga, and As_4_ offers the possibility to perform the growth at much lower temperatures than usual and to accurately control the droplet contact angle and composition at all stages of the growth. Our semiempirical model has proven to be an excellent fit to the experimental Al profile across the heterostructures, providing the means for a rigorous analysis of the interfacial sharpness. The combination of droplet prefilling with Al, thinner nanowires, and lower growth temperatures led to insertions with symmetrical and significantly sharper interfaces, at the limit of the measurement accuracy. The sharpest and most symmetrical insertions were obtained in nanowires with radii of 20–25 nm grown at 350 °C, reaching interface widths as narrow as 2–3 MLs for the whole compositional range of *x*. Our results show that despite the reservoir effect, the interfacial sharpness in VLS-grown axial heterostructures can compete, or even outperform, the sharpness in equivalent thin-film heterostructures grown by well-established methods. In a more general context, we anticipate that our pulsed VLS method is also applicable to other material systems and other nanostructure types than nanowires. Along with sharp heterostructures and precise control of chemical composition, the method combines also features such as monolithic heterogeneous integration and position control, which are crucial for the realization of any hybrid nanotechnology platform.

## Methods

### Growth

All samples were grown by solid-source MBE on Si(111) substrates with a native surface oxide. The first step was the in situ creation of small pinholes in the oxide layer, with controlled size and density, as detailed in refs (32 and 33). In the second step, the substrate temperature was set to *T*_G_ = 550 °C and GaAs nanowires were grown in DCAPE mode.^48^ The third step was the heterostructure growth, which was performed at either the same (*T*_G_ = 550 °C) or a lower temperature. The growth temperatures *T*_G_ refer to the substrate temperature, as measured by an optical pyrometer. All relevant fluxes and pulse durations for growth steps −2 and −3 are provided in the table of Figure S7 in Supporting Information. Ga and Al fluxes (*F*_Ga_ and *F*_Al_, respectively) were calibrated by analyzing the reflection high-energy electron diffraction (RHEED) oscillations during the growth of planar Al_*x*_Ga_1–*x*_As (001) thin films and are here given as growth rates of planar GaAs or AlAs (001) thin films. The A_s4_ flux (*F*_As_), here given as the growth rate of planar GaAs (001), was calibrated using the transition between the As-rich 2 × 4 and the Ga-rich 4 × 2 surface reconstructions on GaAs (001) at given Ga fluxes and substrate temperatures.

The nanowire radius *R*_NW_ can be controlled by adjusting the *F*_As_·τ_As_/*F*_Ga_·τ_Ga_ ratio in step-2 (τ_As_ and τ_Ga_ are the respective pulse durations): the higher the *F*_As_·τ_As_/*F*_Ga_·τ_Ga_ ratio, the smaller the *R*_NW_. This method was used for the systematic variation of *R*_NW_ at *T*_G_ = 550 °C in samples A, D, E, and F (see the table in Figure S7; only sample D exhibited a small deviation in *R*_NW_, probably due to fluctuations in the growth parameters or the surface oxide). To maintain the droplet contact angle at 130–140°, *F*_Ga_ and τ_Ga_ in step-3 were adjusted accordingly for every given *F*_As_ and *R*_NW_. At lower *T*_G_, a higher *F*_Ga_·τ_Ga_ was necessary (see samples A, B, and C in Figure S7). The Al content of each insertion can be finely tuned by varying the Al pulse duration τ_Al_ and/or *F*_Al_. For the majority of samples prepared for this work, *F*_Al_ = 0.1 ML/s and τ_Al_ was varied accordingly to achieve insertions with different Al contents along the nanowires.

Concerning the dot-in-wire heterostructure (see sample G in Figure S7), the nanowire core consists of a GaAs stem grown at 550 °C, followed by two AlAs insertions, separated by 3 nm of GaAs, all grown at 350 °C, and a last GaAs segment grown at 550 °C. All core segments were grown by DCAPE. After the core, the growth was interrupted for 20 min (while ramping down the substrate temperature from 550 to 370 °C) under continuous flux of As_4_ to convert the Ga droplets at the nanowire tips to GaAs. Then, an In_0.3_Al_0.7_As shell was grown in conventional vapor–solid mode (i.e., using continuous fluxes) at 370 °C. At the end, the structure was capped with a 5 nm-thick In_0.3_Ga_0.7_As shell (not shown in the schematic of Figure 6b) to protect the Al-containing shell from oxidation in the air.

### HAADF–STEM: Experimental Measurement of Al-Content Profiles

To quantify the Al composition across an insertion, we analyzed the intensity ratio IR = *I*/*I*_0_ from atomically resolved HAADF-STEM images obtained normal to the nanowire axis along a ⟨1–10⟩ zone axis, using a 200 kV ThermoFisher Talos F200X microscope. The experimental parameters of the HAADF-STEM imaging were as follows: Spherical aberration coefficient: 1.5 mm, Semiconvergence angle: 7.5 mrad, HAADF detector semicollection angles: 62–200 mrad, Defocus: close to −40 nm, Source size (fwhm): 0.1 nm. After subtracting the detector background, *I* is the intensity in regions with Al and *I*_0_ is the intensity taken from a known Al-free region of the GaAs nanowire stem. First, Voronoi tessellation was performed on the images to precisely define the local area occupied by each projected atomic column along ⟨1–10⟩. Integrated intensity contrast profiles running parallel to the nanowire axis were extracted from the Voronoi-tessellated images by averaging the intensities of the Voronoi cells in the direction perpendicular to the nanowire axis. For statistical sampling, 5 nanowires from each growth were analyzed.

The most reliable method to convert experimentally measured IR to Al contents (*x*) is via comparison of IR images/profiles with image simulations, taking into account the experimental STEM/sample parameters and the associated beam broadening. Such image simulations were performed using the STEMsalabim software^64^ using as input a set of rectangular GaAs/Al_*x*_Ga_1–*x*_As/GaAs insertions with different *x* (i.e., 0, 0.25, 0.50, 0.75, and 1) and thicknesses (i.e., 2, 3, and 4 ML, as well as for bulk Al_*x*_Ga_1–*x*_As). A random distribution of Al atoms was considered in each Al_*x*_Ga_1–*x*_As case while the thermal atomic displacements were also taken into account by using the Al, Ga and As Debye–Waller factors at 300 K.^65^ The simulated IR images and the extracted IR profiles across the insertions (for a fixed foil thickness of 40 nm) are shown in Figure S8 in Supporting Information. By assigning the lowest IR value of each simulated profile to the corresponding nominal *x*, and repeating this for different foil thicknesses (same as the nanowire thickness) in the range of 2–80 nm for 2-, 3-, and 4-ML-thick Al_*x*_Ga_1–*x*_As insertions and extending up to 200 nm for the bulk case, IR-to-*x* conversion curves as a function of the insertion and foil thicknesses were produced, as plotted in Figure S9 in Supporting Information. Curves for intermediate *x* values (at a given foil thickness) were estimated via polynomial interpolation.

We note that the IR value of every ML is affected by that of the adjacent MLs on either side, owing to electron cross-scattering. This is especially true for MLs located next to MLs with very different Al contents. This accounts for the shape of the simulated IR profiles, which does not resemble the rectangular shape of the simulated insertion (see Figure S8), and the dependence of IR-to-*x* curves on the insertion thickness (Figure S9). Consequently, the Al content in MLs across very sharp interfaces (0–2 ML thick) can be over- or underestimated as demonstrated in Figure S4, whereas the accuracy improves in thicker interfaces, where compositional grading is slower. Figure S4 shows two abutting insertions, a 3 ML-thick one with *x* = 1.0, and a 2 ML-thick one with *x* = 0.6. As indicated by red arrows, the MLs abutting the GaAs barriers yield a false signal with the one on the left-hand side being strongly overestimated and the one on the right-hand side slightly underestimated. Without us going into a detailed analysis, this example illustrates the effect of cross-scattering being influenced by the neighborhood of the ML, and the limitation it places on the spatial resolution of Al quantification with accuracy. Our image simulations of top-hat compositional profiles with the pertinent material polarity reveal that, at the GA interface, the last (i.e., interfacial) GaAs ML appears to acquire slightly higher intensity, consistent with an artificial Al content. At the AG interface, the last Al_*x*_Ga_1–*x*_As ML appears to have slightly lower intensity consistent with less Al. Therefore, the spatial resolution of the employed chemical analysis method can be defined as 1 ML on either side of each Al_*x*_Ga_1–*x*_As insertion, where the Al composition is artificially affected.

In reality, *x* profiles deviate from the simulated rectangular ones in that *x* is constantly varying across an insertion. Since the actual profile shape is not known beforehand, we opted to employ the simulations for bulk Al_*x*_Ga_1–*x*_As. That is, every ML is treated as if it was bulk Al_*x*_Ga_1–*x*_As with a particular *x* value, which is deduced using the IR-to-*x* conversion curves for bulk Al_*x*_Ga_1–*x*_As. As can be seen in Figure S9, the choice of bulk as a reference instead of a thin insertion is justified as (1) the effect of insertion thickness is considered negligible for ≤0.5, and (2) insertions with *x* > 0.5 are typically thicker than 5 ML, where the bulk approximation works well for the experimentally relevant nanowire (foil) thicknesses. Examples of Al profiles calculated by this method are shown in Figures 1f, 2c, 5d, 6a. Our method was successfully tested against compositional analysis by energy-dispersive X-ray spectroscopy (see Figure S10 in Supporting Information).

In all nanowires, the heterointerface was found to be exactly perpendicular to the long nanowire axis and atomically flat (i.e., without noticeable atomic steps) given that abrupt contrast changes were observed only along the [111] growth direction and never along the [11–2] in-plane direction (i.e., within the (111) MLs), even in the case of thin nanowires. This is consistent with a digital-like ML-by-ML growth mode as observed by in situ TEM.^12^ Of course, the formation of tiny steps extending for just a few atomic distances along the interface cannot be distinguished in STEM images as a projection technique.

### Model Equations

#### *g* Factor

The nanowire geometrical factor *g* = *N*_III_^ML^/*N*_III_^l^, where *N*_III_^ML^ is the total number of group-III atoms in one nanowire ML and *N*_III_^l^ is the total number of group-III atoms in the liquid droplet, quantifies the relative droplet-nanowire geometry,^66,67^ also written as a function of *R*_NW_ and β
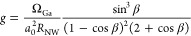
M.1Ω_Ga_ = 1.959 × 10^–2^ nm^3^ is the atomic volume of liquid Ga and *a*_0_ is the GaAs lattice constant.

#### Lambert *W* Function

The Lambert *W* function represents a set of functions defined by *z* = *W*(*z*) *e*^*W*(*z*)^, where *z* can be complex. Since in our case we are dealing with positive real numbers, we only use the principal branch *W*_0_.

#### Model (eq 4 Full Version)


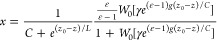
M.2with
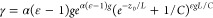


### μ-PL Measurements

For the realization of μ-PL measurements, nanowires were transferred from the as-grown sample onto a Si substrate by gently rubbing the two substrates together, resulting in nanowires that lie flat on their side. The sample is then mounted in a coldfinger cryostat and cooled down with liquid helium to 5 K. For the time-integrated PL measurements we used a Ti/sapphire laser as an excitation pump at a wavelength of 740 nm. The PL is collected with a 100× objective lens (for NIR spectral region) and dispersed into a spectrometer (1800 grooves/mm, energy resolutions down to 30 μeV can be achieved), and then coupled into a charge-coupled device (CCD) camera.

## Supporting Information Available

Measurement of nanowire diameter variations along nanowires. Reproducibility of Al-profiles and nanowire radius in different nanowires. Error bars in Al-content measurements by HAADF-STEM. Effect of electron cross-scattering on Al-profile measurements by HAADF-STEM. Fit parameter C plotted as a function of nanowire radius. Interface parameters L and εg as a function of total Al content and nanowire radius. Table with growth conditions and parameters for all samples in this work. HAADF-STEM image simulations of rectangular Al_*x*_Ga_1–*x*_As insertions with different ML thicknesses. Dependence of HAADF-STEM intensity ratio on nanowire thickness and Al_*x*_Ga_1–*x*_As insertion thickness and composition. Comparison of compositional analysis by HAADF-STEM and energy-dispersive X-ray spectroscopy (EDXS) (PDF)
