# Supplementary material for: At the Limit of Interfacial Sharpness in Nanowire Axial Heterostructures
Source: ACS Nano. 2024 Jul 6;18(32):21171–83. doi: 10.1021/acsnano.4c04172 (PMC11328169; doi:10.1021/acsnano.4c04172)
Supplement: Supplementary file 1 — nn4c04172_si_001.pdf [file nn4c04172_si_001.pdf]

Supporting Information

At the Limit of Interfacial Sharpness in Nanowire Axial Heterostructures

Donovan Hilliard[1, 2,*], Tina Tauchnitz[1, 2], René Hübner[1], Isaak Vasileiadis[3], Athanasios Gkotinakos[3], George Dimitrakopulos[3], Philomela Komninou[3], Xiaoxiao Sun[1], Stephan Winnerl[1], Harald Schneider[1], Manfred Helm[1, 2], and Emmanouil Dimakis[1,*]

[1] *Institute of Ion Beam Physics and Materials Research, Helmholtz-Zentrum Dresden-Rossendorf, Dresden 01328, Germany*

[2] *TUD Dresden University of Technology, Dresden 01062, Germany*

[3] *Department of Physics, Aristotle University of Thessaloniki, Thessaloniki 54124, Greece*

[*] *Corresponding authors*

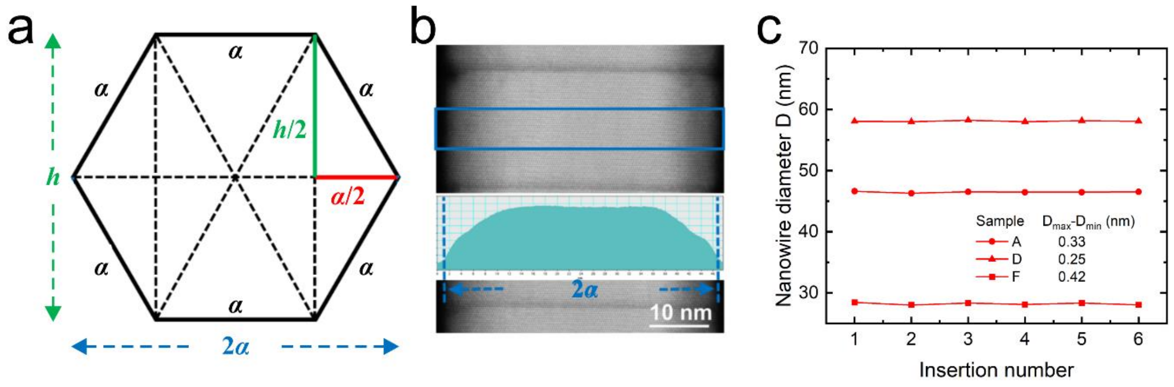

Figure S1: Measurement of diameter variations along a nanowire. (a) Geometry of a hexagonal nanowire. Lengths α and h represent the side and the projected thickness along $\langle 1\bar{1}0 \rangle$ direction, respectively. (b) HRSTEM image of a nanowire. The inset shows the intensity profile obtained from the rectangular area. α can be directly measured from the profile width, whereas $h = \alpha\sqrt{3}$. (c) Representative diameter measurements at the insertion positions along a nanowire (samples A, D, F). The corresponding maximum diameter variations D_{max}-D_{min} (typically less than 0.5 nm) are listed.

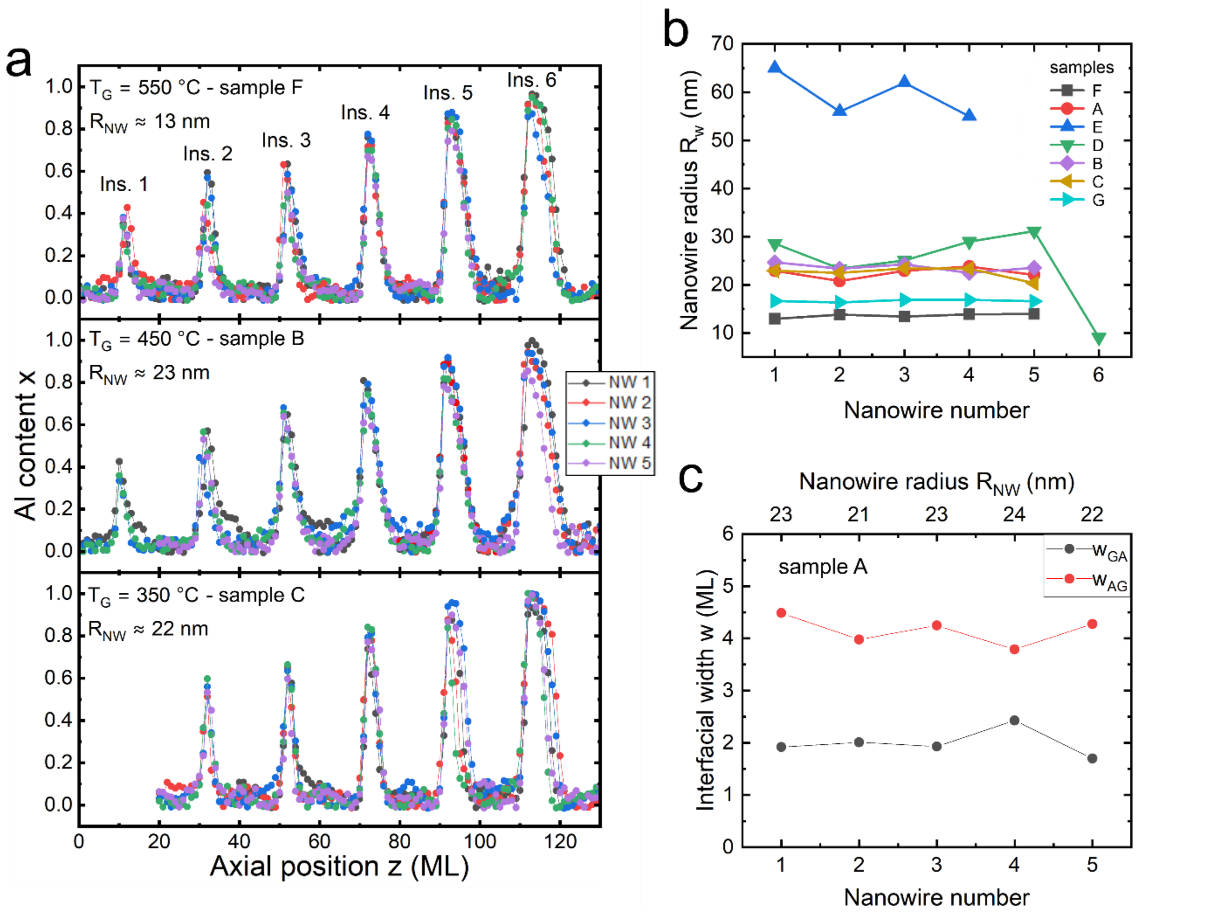

Figure S2. Statistical analysis for the evaluation of the reproducibility of our growth method. The degree of reproducibility can be evaluated by comparing measured Al profiles in

different nanowires of the same sample. (a) Al-content profiles for all insertions (Ins. 1 – 6) in 5 nanowires (NW 1 – 5) from three selected samples (F, B, C). For better visibility and comparison, individual profiles from the same nanowire have been stitched together with a spacing of 20 nm along the x-axis. Most insertions are reasonably-well reproduced in different nanowires of the same sample. Some variations in the total Al content are observed in some cases (mostly for insertions with high x), but the interface sharpness remains reproducible. (b) Variation of the nanowire radius among different nanowires of the same sample (samples A – G). The radius distribution is typically in the range of only a few nm, with very few exceptions (samples E, D). (c) GA and AG interface widths (w_{GA} and w_{AG}, respectively) for insertions with a total Al content of $\alpha \approx$ 3ML in different nanowires of the same sample (A). The width variation is less than 1 ML.

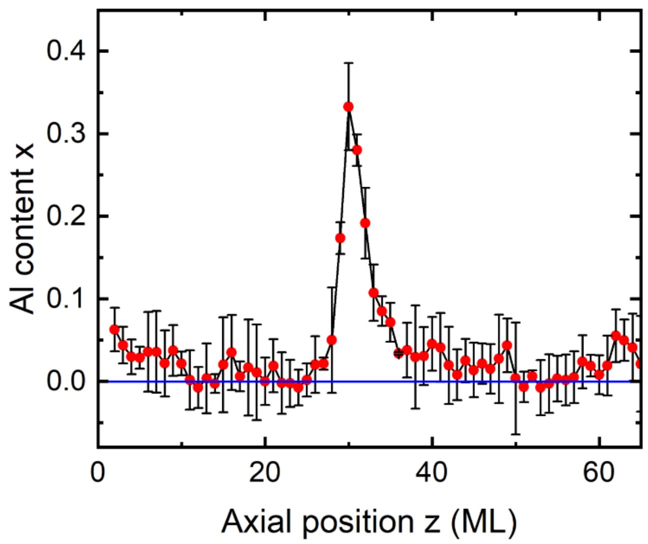

Figure S3: Error bars in Al-content measurements. The Al profile around the first $Al_xGa_{1-x}As$ insertion (Al pulse of 8 s and 0.05 ML/s) in Fig. 1e including error bars. Typically, the error in x is in the range of ± 0.05.

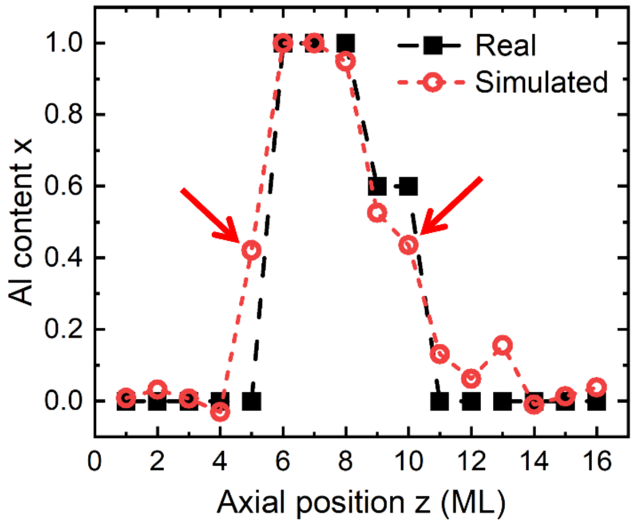

Figure S4: Demonstration of the contribution of electron cross-scattering into abutting MLs. Two $Al_xGa_{1-x}As$ profiles are shown for 40-nm-thick nanowires (R_{NW} = 20 nm). Black squares connected by a dashed line represent a profile containing the real Al content present in the MLs of the simulated supercell, chosen manually as follows: from left to right x = 1.0, 1.0, 1.0, 0.6, 0.6. The red profile is the output of the HAADF image simulation including the influence of electron cross-scattering. Red arrows highlight two extreme cases in which cross-scattering yields false Al contents in Al-containing MLs (right arrow) and even creates a completely false Al signal in an Al-free ML (left arrow).

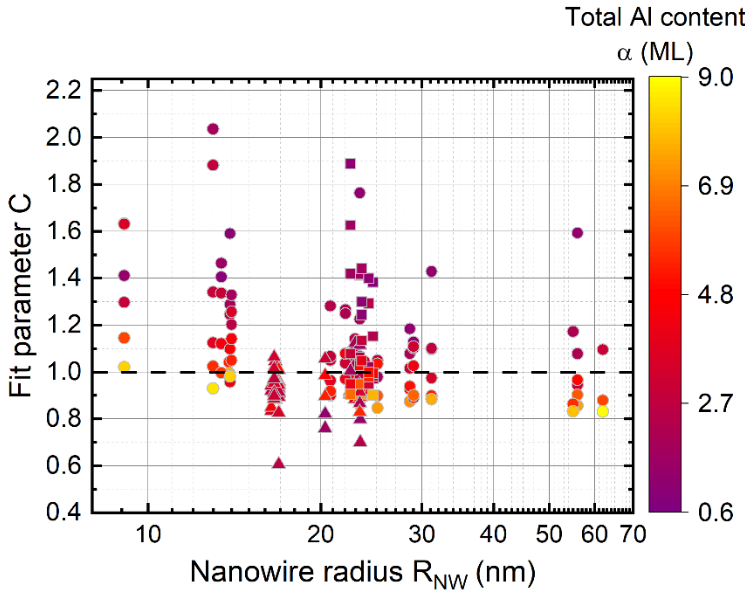

Figure S5: Fit parameter C plotted as a function of nanowire radius R_{NW}. Circles, squares, and triangles represent insertions grown at 550, 450, and 350 °C, respectively. The color gradient is a scale for the total Al content α. The dashed line is a guide to the eye highlighting C = 1.

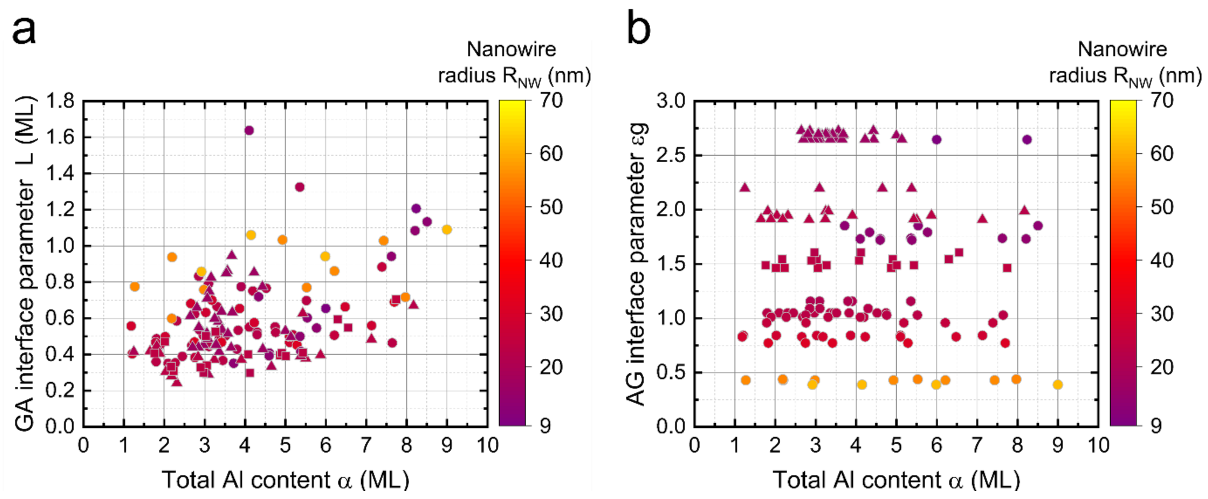

Figure S6: Comparisons of the interface parameters L and εg to both the total Al content α and the nanowire radius R_{NW} demonstrating the behavior of the profile slopes. (a) GA interface parameter L plotted as a function of the total Al content α with a color gradient scale for R_{NW}. (b) As the plot in (a), but here for the AG interface parameter εg. Circles, squares, and triangles represent insertions grown at 550, 450, and 350 °C, respectively.

Sample ID	T_G (°C)	τ_{Ga} (s)	τ_{As} (s)	τ_{Al} (s)	τ_{As-1} (s)	F_{Ga} (ML/s)	F_{As} (ML/s)	F_{Al} (ML/s)	R_{NW} (nm)
A	550	5	6			0.11	0.22		22
	550	5	6	8,10,12,8,10,12	12,12,12,12,12,12	0.11	0.22	0.05,0.05,0.05,0.10,0.10,0.10	
B	550	5	6			0.11	0.22		23
	450	12	6	12,12,12,12,15,15	12,12,12,12,15,15	0.11	0.22	0.10	
C	550	5	6			0.11	0.22		22
	350	16	6	2,4,6,10,16,24	12,12,12,12,15,15	0.11	0.22	0.10	
D	550	2	4			0.09	0.22		9 – 30
	550	2	4	2,4,6,10,16,24	12,12,12,12,15,15	0.09	0.22	0.10	
E	550	10	6			0.11	0.22		55 – 65
	550	10	6	2,4,6,10,16,24	20,20,20,20,25,25	0.11	0.22	0.10	
F	550	5	4			0.01	0.30		13
	550	8	4	2,4,6,10,16,24	12,12,12,12,15,15	0.01	0.22	0.10	
G	550	5	4			0.01	0.30		16
	350	18	6	8,8	15,15	0.09	0.22	0.20	

Figure S7: Table containing the growth conditions and parameters for all samples in this work. Gray- and yellow-shaded rows correspond to growth steps -2 and -3, respectively. Shown are the growth temperature (T_G), the nanowire radius (R_{NW}), and the flux and duration of Ga (F_{Ga}, τ_{Ga}), Al (F_{Al}, τ_{Al}), and As_4 (F_{As}, τ_{As}) pulses. τ_{As-1} corresponds to the duration of the first As_4 pulse after Al.

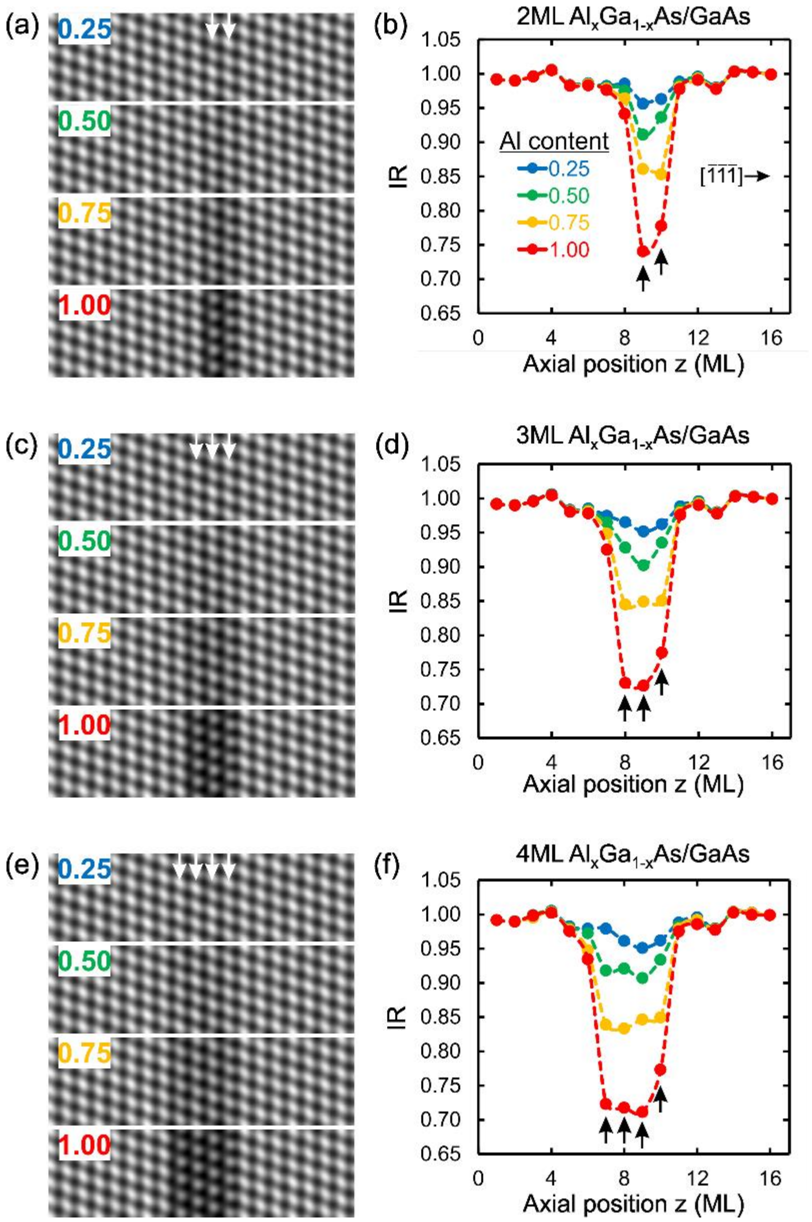

Figure S8: HAADF-STEM image simulations of rectangular $Al_xGa_{1-x}As$ insertions with different ML thicknesses. (a), (c), and (e) are 2, 3, and 4 ML, respectively, and, for each case, a set of four Al contents is illustrated (x = 0.25 (blue), 0.50, (green), 0.75 (yellow), and 1 (red)). The plots in (b), (d), and (f) are the respective IR profiles extracted from (a), (c), and (e). Black arrows denote the locations of the inserted MLs.

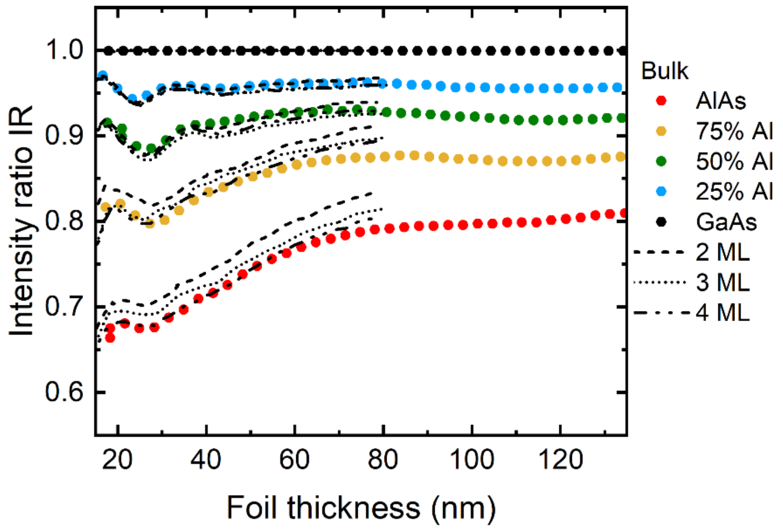

Figure S9: IR dependence on nanowire (foil) thickness for the case of bulk $Al_xGa_{1-x}As$ (colored data points) and the corresponding case of 2-, 3-, and 4-ML-thick $Al_xGa_{1-x}As$ insertions (dash, dot, and dash-dot lines, respectively).

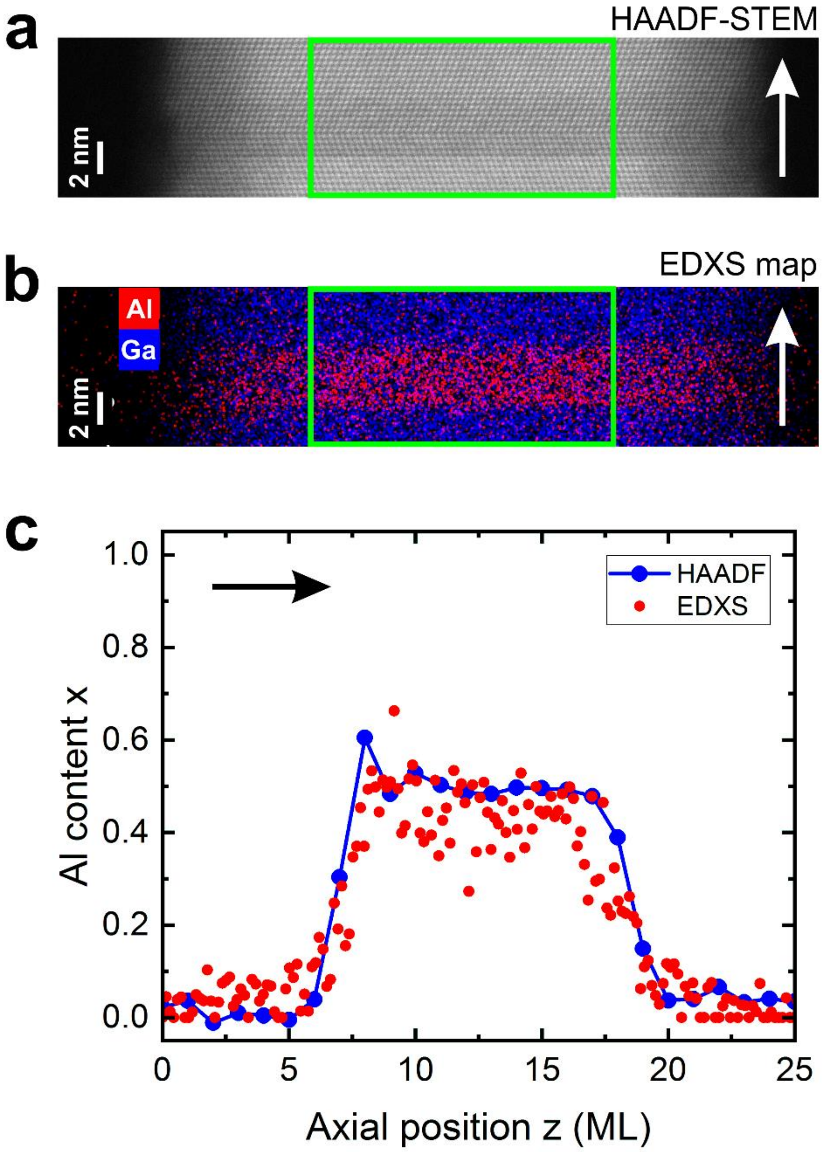

Figure S10. Comparison of compositional analysis by HAADF-STEM imaging and EDXS. (a) High-resolution HAADF-STEM image. (b) EDXS compositional map: red for Al and blue for Ga. (c) Extracted Al-content profiles from the framed areas with green rectangles in (a) and (b). Both methods resolved the rectangular shape of the insertion and resulted in similar plateau values for the Al content and similar insertion widths. In contrast to HAADF-STEM, though, EDXS failed to resolve the local (1 ML-thick) peak of Al content at the beginning of the insertion owing to the lack of atomic resolution. The block arrows in all panels indicate the growth direction.
